# Is frontoparietal electroencephalogram activity related to the level of functional disability in patients emerging from a minimally conscious state? A preliminary study

**DOI:** 10.3389/fnhum.2022.972538

**Published:** 2022-09-29

**Authors:** Wanchun Wu, Chengwei Xu, Xiyan Huang, Qiuyi Xiao, Xiaochun Zheng, Haili Zhong, Qimei Liang, Qiuyou Xie

**Affiliations:** Joint Research Centre for Disorders of Consciousness, Department of Rehabilitation Medicine, Zhujiang Hospital, Southern Medical University, Guangzhou, Guangdong, China

**Keywords:** emergence from minimally conscious state, disability rating scale, EEG, frontoparietal region, functional connectivity

## Abstract

**Objective:**

When regaining consciousness, patients who emerge from a minimally conscious state (EMCS) present with different levels of functional disability, which pose great challenges for treatment. This study investigated the frontoparietal activity in EMCS patients and its effects on functional disability.

**Materials and methods:**

In this preliminary study, 12 EMCS patients and 12 healthy controls were recruited. We recorded a resting-state scalp electroencephalogram (EEG) for at least 5 min for each participant. Each patient was assessed using the disability rating scale (DRS) to determine the level of functional disability. We analyzed the EEG power spectral density and sensor-level functional connectivity in relation to the patient’s functional disability.

**Results:**

In the frontoparietal region, EMCS patients demonstrated lower relative beta power (*P* < 0.01) and higher weighted phase lag index (wPLI) values in the theta (*P* < 0.01) and gamma (*P* < 0.01) bands than healthy controls. The frontoparietal theta wPLI values of EMCS patients were positively correlated with the DRS scores (*r*_*s*_ = 0.629, *P* = 0.029). At the whole-brain level, EMCS patients only had higher wPLI values in the theta band (*P* < 0.01) than healthy controls. The whole-brain theta wPLI values of EMCS patients were also positively correlated with the DRS scores (*r*_*s*_ = 0.650, *P* = 0.022). No significant difference in the power and connectivity between the frontoparietal region and the whole brain in EMCS patients was observed.

**Conclusion:**

EMCS patients still experience neural dysfunction, especially in the frontoparietal region. However, the theta connectivity in the frontoparietal region did not increase specifically. At the level of the whole brain, the same shift could also be seen. Theta functional connectivity in the whole brain may underlie different levels of functional disability.

## Introduction

Severe brain injury may lead to prolonged impairment of consciousness, that is, disorders of consciousness (DoC). Frequently, the recovery trajectory of DoC is a vegetative state or “unresponsive wakefulness syndrome” (VS/UWS) (Laureys et al., 2010), minimally conscious state (MCS), and emergence from MCS (EMCS, also called confusional state) (Giacino et al., 2002).

When regaining consciousness (able to use objects or communicate functionally) (Giacino et al., 2002), 98% of EMCS patients have cognitive impairment and are largely dependent on caregivers for basic activities of daily living (Nakase-Richardson et al., 2009; Murphy, 2018; Bodien et al., 2020). These sequelae of brain injury create great challenges for evaluation, therapy, and prognosis. However, Bodien et al. observed that EMCS patients had a large spread in the total scores for the disability rating scale (DRS), which is used to assess disability outcomes for severely brain-injured patients (Rappaport et al., 1982; Rappaport, 2005), and categorized several levels of dysfunction ranging from VS to moderate disability (Bodien et al., 2020). This implies that some EMCS patients could return to society, while others remained unable to take care of themselves. However, the cause of EMCS patients having different levels of functional disability after regaining consciousness remains unclear. Objective measurements are needed to assess cortical function, which may help to explain this phenomenon.

Several differential features between EMCS and MCS or VS/UWS have been reported (Rodriguez Moreno et al., 2010; Lesenfants et al., 2016; Aubinet et al., 2018). The degree of impairment of the frontoparietal network observed using positron emission tomography, magnetic resonance imaging, and electroencephalography is often used to determine the level of consciousness in patients (Fernández-Espejo et al., 2012; Stender et al., 2015; Wu et al., 2020). EMCS patients with a higher level of consciousness demonstrate more preserved metabolism in the frontoparietal network than VS/UWS and MCS patients, while they have lower regional cerebral glucose metabolism rates than healthy controls (HCs; the maximum difference is in the frontoparietal cortex) (Thibaut et al., 2012; Stender et al., 2015; Bodart et al., 2017). The frontoparietal network comprises two subnetworks as follows: the default mode network (DMN), which is involved in internal awareness and self-reflection, and the executive control network (ECN), which processes external stimuli and mediates attention (Vogt and Laureys, 2005; Vanhaudenhuyse et al., 2011; Buckner and DiNicola, 2019). The connectivity between the two subnetworks is correlated with metabolic activity and has been demonstrated to clearly characterize EMCS patients and indicate a neural correlate of consciousness (Di Perri et al., 2016). Ongoing impairments within these subnetworks may result in various levels of dysfunction. Clinically, EMCS patients experience confusion and perceptual disturbances (Nakase-Richardson et al., 2009; Bodien et al., 2020). Thus, we hypothesized that there may be impairment in the frontoparietal function of patients and that this may be associated with functional disability.

In this study, we aimed to verify the hypothesis that electroencephalogram (EEG) measurements of the frontoparietal area may be helpful in characterizing EMCS patients and to establish an objective method to quantify the degree of functional disability. We used EEG measurements to preliminarily explore differences in frontoparietal cortical function between EMCS patients and HCs, and examined the relationship between patients’ EEG activities and their levels of disability.

## Materials and methods

### Participants

In total, 12 EMCS patients (mean age 47.2 ± 14.4 years, five men) and 12 HCs (mean age 46.0 ± 12.8 years, seven men) were recruited for this preliminary study. The EMCS patient assessments were confirmed using the Coma Recovery Scale-Revised (CRS-R), and patients had one or both of the following behaviors: functional interactive communication or functional use of two different objects (Giacino et al., 2002, 2004). The patients enrolled in the study did not receive any treatment with sedatives or psychostimulants, such as clonazepam, midazolam, zolpidem, or modafinil for at least 2 days before data collection. Patients with a history of epilepsy or other neuropsychological diseases were excluded from this study. Written informed consent to participate in this study was obtained from the HCs and legally authorized representatives of patients. The ethics committee of Zhujiang Hospital approved all aspects of the study.

### Behavioral assessment

Before the recording session, each patient was assessed three times by two experienced raters with the CRS-R within a week and on the last day of EEG recording (Bodart et al., 2017). The best result was maintained as a behavioral diagnosis. The DRS is designed to assess functional disability in patients with brain injury. The scores range from no disability (0) to death (30), and have ten disability categories. Higher scores reflect poorer functioning (Rappaport et al., 1982; Rappaport, 2005). In this study, the DRS score was observed within 3 days of EMCS diagnosis (Bodien et al., 2020).

### Electroencephalogram recording and preprocessing

For each participant, resting-state EEG was recorded for at least 5 min (completed between 30 and 85 trials, 10 s per trial). During recording, the patients sat in a wheelchair with their eyes open, and the standard CRS-R arousal facilitation protocol was used to maintain an arousal state. The controls were asked to relax, be awake with their eyes open, and not engage in any specific activity. The brain activity of the participants was recorded using a 66 channel system (SynAmps2TM 8500; Neuroscan, USA) with a 2,500-Hz sampling frequency, followed by the International 10–20 System. The machine used Ag/AgCl pin electrodes with band-pass filtering at a direct current of 1,000 Hz. During recording, the electrode impedance was maintained at <5 kΩ. To objectively assess the open state of the eyes, we measured vertical eye blink-related activity by electrooculography (EOG) similar to that used in a previous study (Chennu et al., 2014; Supplementary Figure 1).

Electroencephalogram preprocessing was conducted using the EEGLAB toolbox (13_0_0b) in MATLAB (version 2013b; MathWorks Inc., Natick, MA, USA). The EEG data were downsampled to 500 Hz and filtered between 0.5 and 45 Hz. The EEG signals were divided into epochs of 10 s. Artifacts derived from nearby muscle activity and eye movements were eliminated using independent component analysis. Epochs containing evident artifacts were manually removed by visually inspecting the data of each participant. Epochs with activity exceeding ± 100 μV were rejected using a semi-automated procedure, and the artifact-free signals were averaged. After preprocessing, the data of the participants were retained between 22 and 56 trials. Therefore, we selected 22 trials for each participant separately to match the trial numbers across the groups for further analysis.

### Power spectral analysis

The Welch method was used to compute the power spectral density (PSD, with a 50% overlap between 1-s Hamming windowed segments) for each signal epoch. The absolute powers of the delta (0.5–4 Hz), theta (4–8 Hz), alpha (8–13 Hz), beta (13–30 Hz), and gamma (30–45 Hz) bands were estimated. To normalize and compute the relative power, the absolute PSD values at each frequency band relative to the total power across the entire frequency spectrum were calculated. To verify our hypothesis, the relative power for each band was averaged across channels in both the frontoparietal region and whole brain: frontoparietal region (frontal: Fp1, Fp2, FPz, AF3, AF4, Fz, F1, F2, F3, F4, F5, F6, F7, F8, parietal: CPz, CP1, CP2, CP3, CP4, CP5, CP6, Pz, P1, P2, P3, P4, P5, P6).

### Functional connectivity analysis

A functional connectivity analysis was performed using the weighted phase lag index (wPLI). The wPLI measure is more robust and partially invariant to volume conduction in phase relationships (Peraza et al., 2012). wPLI is defined as the phase difference between two signals from the frontal and parietal regions, weighted by the magnitude of the imaginary component of the cross-spectrum within the band of interest. Calculations were performed according to a previous study (Vinck et al., 2011). The values of wPLI range from 0 to 1, with 1 indicating strong functional connectivity and 0 indicating no connectivity. The mean connectivity of the frontoparietal region and whole brain was measured for all frequency bands in each group.

### Statistical analysis

Due to the non-normality of the data, we used a non-parametric permutation test to identify the differences in EEG relative power and the wPLIs within the frontoparietal region and the whole brain between EMCS patients and HCs in the frequency ranges (number of permutations > 1,000; *P* < 0.05). Bonferroni correction was used for multiple comparison of the correction for each frequency band (*n* = 5). Pairwise comparisons of connectivity between every electrode were performed using two-sample *t*-tests, and the network-based statistic was performed after multiple comparisons using the graph theory network analysis toolbox (Wang et al., 2015). An edge *p*-value of 0.01 was set for the t matrix. Next, statistical significance was estimated with 5,000 permutation tests and a family-wise error corrected level of *P* < 0.05. Therefore, we performed Spearman’s correlations to separately estimate the relationship between DRS scores and significant EEG parameters in defined regions of interest in EMCS patients.

## Results

No significant differences in age and sex were observed between EMCS patients and HCs (*P* > 0.05). The details of EMCS patients, including etiology, post-injury (months), lesions, cranioplasty, EMCS behaviors, CRS-R sub-scale, CRS-R total scores, and DRS total scores are presented in Table 1. The DRS total scores were categorized as follows: moderately severe disability (scores 7–11), severe disability (scores 12–16), and extremely severe disability (scores 17–21). None of the enrolled patients demonstrated functional object use or communication in the CRS-R. Regarding scores, one patient had 11, 13, and 18, two had 21, three had 20, and four had 19. Furthermore, we observed no significant difference between the average EOG activity in the first and second halves of the recording in EMCS patients (*P* > 0.05; Supplementary Figure 1). The EEG data between patients who received cranioplasty and those who did not revealed no significant differences in the five frequency bands (all *P*-values > 0.05; Supplementary Figure 2).

**TABLE 1:** Patients’ demographic characteristics.

Patient No./gender/age (years)	Etiology	Post-Injury (months)	Lesions (CT or MRI)	Cranioplasty	EMCS behaviors	CRS-R sub-scores	CRS-R total scores	DRS
1/F/47	Hemorrhage	11	Left frontal lobe, Right cerebellum	Left frontal lobe	FOU	4-5-6-2-1-2	20	19
2/M/34	TBI	8	Bilateral frontal lobe, brain stem	-	FOU	2-3-6-3-0-2	16	19
3/M/59	Hemorrhage	13	Left frontal, temporal lobes, basal ganglia	Left frontal, temporal parietal lobe	FOU	2-3-6-2-0-2	15	13
4/F/67	Hemorrhage	3	Right basal ganglia	-	FOU	2-3-6-2-0-2	15	19
5/F/37	Hemorrhage	11	Left occipital lobes	Left frontal, temporal, parietal lobe	FOU	3-5-6-3-1-2	20	11
6/F/38	TBI	4	Left frontal lobe, brain stem	-	FIC	3-5-2-2-2-2	16	20
7/F/33	Hemorrhage	11	Right temporal lobe	-	FOU	1-3-6-2-0-2	14	20
8/F/69	Hemorrhage	9	Bilateral frontal, parietal sub-cortical	-	FOU	2-3-6-2-0-2	15	19
9/M/34	Hemorrhage	6	Left basal ganglia, brain stem	-	FIC	4-5-2-1-2-2	16	21
10/M/40	Anoxia	7	Diffuse anoxic-ischemic	-	FIC	3-0-2-3-2-2	12	18
11/M/40	Hemorrhage	3	Right thalamus	-	FOU	3-1-6-1-0-2	13	21
12/F/68	TBI	6	Diffuse demyelination	-	FOU	2-3-6-1-0-2	14	20

FOU, functional object use; FIC, functional interactive communication; CRS-R, coma recovery scale-revised; Subscales score of CRS-R indicating the assessment of auditory, visual, motor, verbal, communication functions and arousal; DRS, Disability Rating Scale; TBI, traumatic brain injury.

**FIGURE 1:**
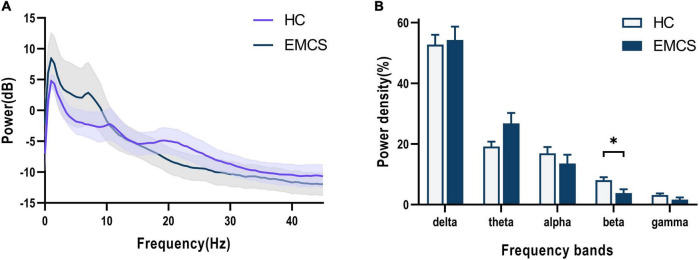
Frontoparietal changes in the EEG power spectra in the emergence from minimally conscious state (EMCS) group compared with the healthy control (HC) group. **(A)** The average absolute EEG power in HCs (purple line) and EMCS patients (blue line). The Y-axis represented the log-transformed power. **(B)** The relative power in five frequency bands. A marked decrease in the relative power in beta frequencies was observed in the EMCS group compared with the power in the HC group (*P < 0.01, after Bonferroni correction for frequency bands). The data were expressed as the means ± SEM.

**FIGURE 2:**
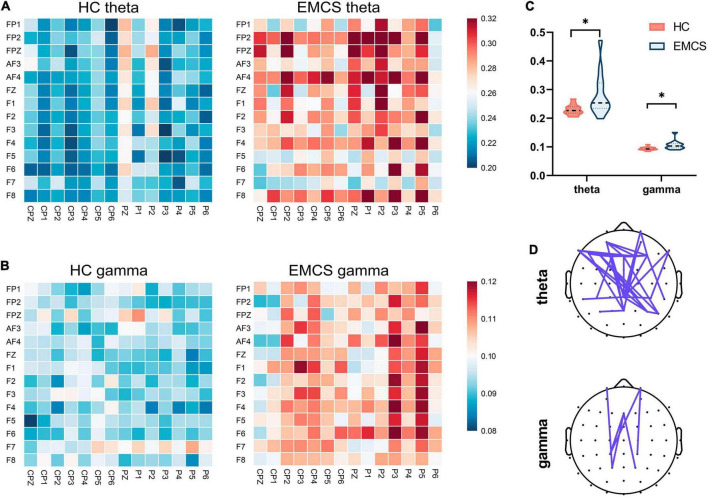
Frontoparietal weight phase lag index (wPLI) among healthy control (HC) group and emergence from minimally conscious state (EMCS) group. **(A)** Heatmaps of averaged wPLI values at rest for theta band between the groups. **(B)** Heatmaps of averaged wPLI values at rest for gamma band. The wPLI values were implemented Fisher-Z transformed. **(C)** Violin plot of wPLI values between frontal and parietal areas in theta and gamma bands (*P < 0.01, after Bonferroni correction). **(D)** The top panel shows significantly altered connectivities between the two groups. The purple line means significantly increased connectivity in EMCS patients.

In terms of the frontoparietal region, the EMCS patients demonstrated lower relative power in the beta band than the HCs (*P* = 0.007; Figures 1A,B). Furthermore, the wPLI increased in the theta (*P* = 0.008) and gamma (*P* = 0.004) bands in this region in EMCS patients. Figures 2A,B present the average functional connectivity of channels in the frontoparietal region. Fisher-Z transformation was used to normalize the distribution of the values. Figure 2C depicts the mean ± SEM of the wPLI values in the frontoparietal region. After multiple comparisons of each connectivity value, the significantly altered connectivity was consistent with the average connectivity findings (Figure 2D). Compared with the HC group, the increased connectivity of the theta band occured between the frontal and parietal regions and within these regions in the EMCS patients. Increased connectivity of the gamma band was mostly observed between the frontal and parietal regions in the EMCS group. A significant correlation was identified between theta functional connectivity and the total DRS score in the frontoparietal area. The wPLI in the theta band was positively correlated with DRS scores (*r*_s_ = 0.629, *P* = 0.029). However, gamma wPLI and beta relative power did not correlate with the DRS scores (*r*_s_ = –0.047, *P* = 0.885; *r*_s_ = –0.445, *P* = 0.147).

**FIGURE 3:**
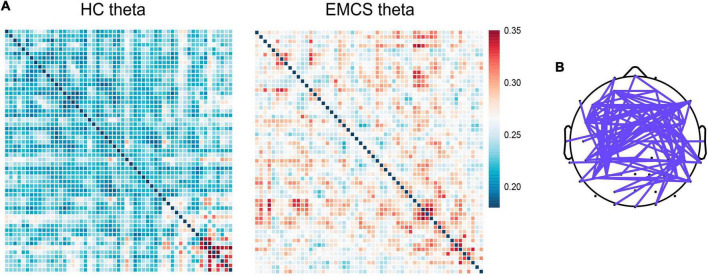
Global weight phase lag index (wPLI) among healthy control (HC) group and emergence from minimally conscious state (EMCS) group. **(A)** Heatmaps of averaged wPLI values at rest for theta band between the groups. The wPLI values were implemented Fisher-Z transformed. **(B)** The top panel shows significantly altered connectivities between the two groups. The purple line means significantly increased connectivity in EMCS patients.

**FIGURE 4:**
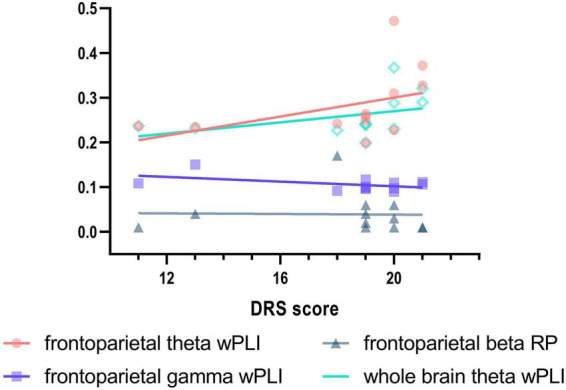
Correlation between Disability Rating Scale (DRS) scores and EEG activity in EMCS patients. Spearman’s correlations between DRS and frontoparietal theta wPLI (*r*_s_ = 0.629, *P* = 0.029), frontoparietal gamma wPLI (*r*_s_ = –0.047, *P* = 0.885), frontoparietal beta relative power (*r*_s_ = –0.445, *P* = 0.147), and whole brain theta wPLI (*r*_s_ = 0.650, *P* = 0.022) in EMCS patients. Linear regression was used to generate fitted lines for each data set.

At the whole-brain level, relative power did not differ between the two groups. Compared with the HCs, the EMCS patients had increased wPLI values in the theta band (*P* = 0.004). Figure 3A presents the average functional connectivity of channels across the brain. After multiple comparisons of each connectivity value, the significantly altered connectivity was consistent with the average connectivity findings (Figure 3B). The theta wPLI value in the whole brain revealed a positive correlation with DRS scores (*r*_s_ = 0.650, *P* = 0.022, Figure 4).

To clarify whether such EEG changes were specifically altered in the frontoparietal region, we also compared measures in the whole brain and frontoparietal region in the EMCS group. No significant differences in power and functional connectivity were observed between the two groups at any frequency (Supplementary Figure 3).

## Discussion

Emergence from minimally conscious state is a late stage in the typical recovery of DoC patients with severe brain injury etiologies. Patients at this stage have different levels of dysfunction. Once patients regain consciousness, clinicians often focus on treating cognitive impairment, lack of speech, and motor dysregulation (Lancioni et al., 2014). Therefore, a reliable and objective assessment is necessary to characterize this population. Our study evaluated patients who experienced EMCS using EEG activity and analyzed the effects of EMCS on functional disability. This study had three main preliminary findings as follows: EMCS patients had varying degrees of severe disability; EMCS patients still had abnormal EEG activity, especially in the frontoparietal region; and theta wPLI correlated with functional disability in EMCS at the whole-brain level.

In terms of functional disability, EMCS patients were associated with severe disability on the DRS, ranging from moderate to extremely severe disability. They demonstrated a restricted range of cognition and behavior. The DRS scores were expected and in line with those of earlier investigations (Murphy, 2018; Bodien et al., 2020).

Additionally, the relative beta power was lower in the frontoparietal region in EMCS patients than in HCs, which is inconsistent with the “ABCD” model suggested by Schiff that EMCS patients have dominant peaks in higher frequency ranges (i.e., 15–40 Hz) (Schiff, 2016). There could be three possible reasons for this inconsistency. First, lower beta band may appear in EMCS patients in contrast to that in HCs. EMCS patients have two EEG types in the ABCD model, the first characterized by oscillations at 5–9 Hz and 15–40 Hz (C-type) and the second characterized by oscillations at 8–12 Hz and 15–40 Hz (D-type or healthy). C-Type is produced by burst central thalamic activity, while D-type is driven by tonic firing thalamic activity. Insufficient firing and moderate deafferentation in thalamocortical connectivity may reduce the beta power (Llinás et al., 2005). However, the beta power may be relatively preserved when compared to other patients with DoC or unrecovered patients with severe brain injury, who have quiescent central thalamic activity and more severe deafferentation. Second, EMCS patients could have local electrophysiological abnormalities even in the D-type spectrum, such as an increased delta to alpha power ratio (Shah et al., 2017b; Edlow et al., 2021). A previous study has reported that regional beta power is reduced in mild traumatic brain injury patients who have functional impairment (Zhang et al., 2020). Similarly, patients who recover full consciousness have deficits in attention owing to the suppression of frontal beta power (Shah et al., 2017a). Third, the observed pattern may not fit the ABCD model. According to a recent study, some EEG activity (10.6%) in patients with acute traumatic brain injury with DoC did not correspond to any ABCD type. Though not all data contained peak combinations from the ABCD model, the dataset could still significantly predict the recovery of consciousness (Frohlich et al., 2022). Beta oscillations are associated with motor function and various cognitive functions, including predictive coding, working memory, perception, and emotion (Roa Romero et al., 2015; von Lautz et al., 2017; Chang et al., 2018; Betti et al., 2021; Schubring and Schupp, 2021). The decrease in the beta power in EMCS patients may indicate reduced cortical function.

In the connectivity analysis, the EMCS patients demonstrated higher theta wPLI values both in the frontoparietal region and the whole brain, and higher wPLI values in gamma bands within the frontoparietal region than the HCs. Cavaliere et al. (2018) also observed that patients have predominant posterior theta activity. Theta activity is generally considered to reflect fundamental cognitive functions (Biel et al., 2022; Cowley et al., 2022). An association between increased theta connectivity and cognitive dysfunction has been reported in some diseases such as Alzheimer’s disease, Parkinson’s disease, and schizophrenia (Andreou et al., 2015; Iyer et al., 2020; Yan et al., 2021). Increased gamma connectivity can facilitate synchronization during cognitive processing (Başar, 2013). The EMCS patients who demonstrate higher theta and gamma connectivity may have difficulty maintaining basic cognition in a resting state. Thus, they need to apply more neural resources to integrate information. Gamma oscillations originating from inhibitory interneurons by spreading gamma-aminobutyric acid (GABA) are considered an efficient mechanism for functional connectivity (Fingelkurts et al., 2004; Jafari et al., 2020). Theta activity also requires GABAergic and cholinergic input (Başar, 2013). A high level of energy expenditure in the theta and gamma bands at rest may implicate ongoing neural dysfunction in neurotransmitter systems such as GABA.

Our results revealed that increased theta band wPLI values in the frontoparietal region were positively correlated with total DRS scores. However, this correlation was also observed at the whole-brain level. Interestingly, EMCS patients had similar power and connectivity changes between the frontoparietal region and the whole brain, suggesting that theta connectivity increases are not specific to the frontoparietal regions. However, the current results are insufficient to support the hypothesis that abnormalities in the frontoparietal region affect dysfunction. The reasons for similar changes in patients need further evaluation. Additionally, the different effects of beta power and gamma connectivity within the frontoparietal region might be driven by the differences between the frontoparietal region and the whole brain in HCs.

Nevertheless, theta wPLI correlated with functional disability at the whole-brain level in the EMCS. This suggests that increased theta functional connectivity may indicate severe dysfunction in EMCS patients. The DRS includes the assessment of physical and cognitive items, reflecting functional disability in patients with severe brain injury (Rappaport et al., 1982). Alterations in theta oscillations are not only linked with cognition but also with impulse control disorders (Zhu et al., 2019). Therefore, the correlation we observed has a plausible biological interpretation. Due to the small sample size, we did not directly link the EEG results to specific DRS items. In fact, most EMCS patients have cognitive impairments (Bodien et al., 2020). Physical disorders are a frequent complication of severe brain injury (Thibaut et al., 2015). Further studies should directly assess cognition in EMCS patients to verify the relationship between functional details and EEG activity.

Several limitations of this study should be noted. First, the broad range of etiologies and multiple structural lesions were not considered. Patients with traumatic brain injury often experience greater rehabilitation efforts (Colantonio et al., 2011). Second, this population was heterogeneous (Marino and Whyte, 2022). Complex stratification in our study could have led to inaccurate results because of the lower number of patients in the experimental group. If available, it may be more appropriate to select patients with brain injury having the same etiology and history of no DoC as controls, which may reduce the impact of brain damage on the EEG results. Third, although we preliminarily observed frontoparietal activity in this study as a previous study has reported one of the maximum differences between EMCS patients and healthy individuals in this region (Cavaliere et al., 2018), the spatial resolution of EEG is limited. Multimodal neuroimaging methods can be used to analyze the correlation between brain function (e.g., ECN and DMN source connectivity) and functional disability, which has a higher spatial and temporal resolution and may provide more meaningful information.

## Conclusion

Emergence from minimally conscious state patients with a higher consciousness level could experience ongoing neural dysfunction, especially in the frontoparietal region. Theta functional connectivity at the whole-brain level may be a mechanism for the different levels of functional disability in this population. Exploration of brain features may contribute to a more detailed characterization of EMCS patients, and sustained treatment of such patients should be considered. In the future, patients could undergo individual cognitive evaluation and rehabilitation programs.

## Data availability statement

The datasets presented in this article are not readily available because the data also forms part of an ongoing study. Requests to access the datasets should be directed to the corresponding author QXe.

## Ethics statement

The studies involving human participants were reviewed and approved by Zhujiang Hospital, Southern Medical University. The patients/participants provided their written informed consent to participate in this study.

## Author contributions

WW contributed to the conception and design of the study, performed the statistical analysis, and wrote the first draft of the manuscript. WW, CX, QXa, XZ, XH, HZ, and QL organized the database. CX, XH, QXa, and XZ wrote sections of the manuscript. QXe contributed to the conceptualization, funding acqusition, resources, supervision, and writing–review and editing. All authors contributed to the manuscript revision, read, and approved the submitted version.

## Abbreviations

CRS-R: coma recovery scale-revised
DMN: default mode network
DRS: disability rating scale
ECN: executive control network
EEG: electroencephalogram
EMCS: emergence from minimally conscious state
EOG: electrooculography
GABA: gamma-aminobutyric acid
HC: healthy control
MCS: minimally conscious state
PSD: power spectral density
VS/UWS: vegetative state/unresponsive wakefulness syndrome
wPLI: weighted phase lag index.
