# Supplementary material for: Is frontoparietal electroencephalogram activity related to the level of functional disability in patients emerging from a minimally conscious state? A preliminary study
Source: Front Hum Neurosci. 2022 Sep 29;16:972538. doi: 10.3389/fnhum.2022.972538 (PMC9556633; doi:10.3389/fnhum.2022.972538)
Supplement: Supplementary file 1 [file Data_Sheet_1.pdf]

Supplementary Material

1 Supplementary Figures

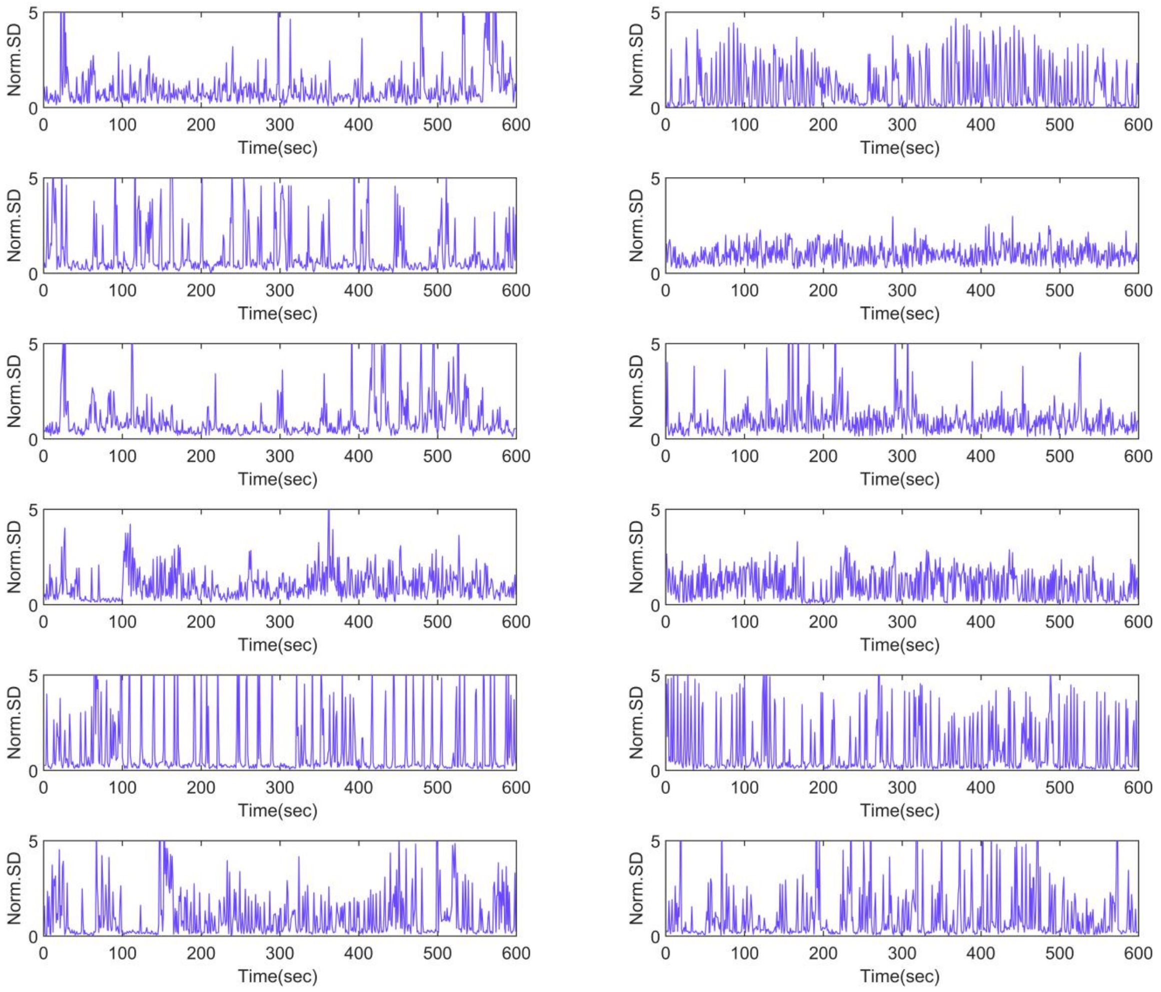

Supplementary Figure 1. Eye-movement related activity and calculated standard deviation. The vertical electrooculographic (EOG) channels of all patients with 1–3 Hz were filtered to focus on eye-movement related activity, and then their standard deviations (SD) within a 1-second non-overlapping sliding window over time were calculated and normalised by the average SD over all such windows. There was no significant difference between average activity in the first half of the recording session and the second half in EMCS patients (using a paired t-test, P=0.23).

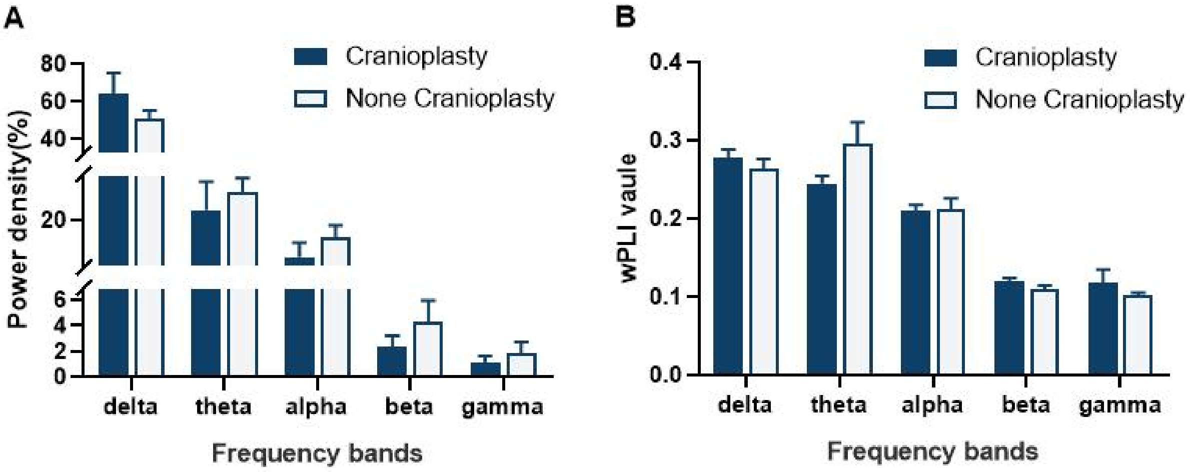

Supplementary Figure 2. The relative power and wPLI value between cranioplasty and none cranioplasty patient. There was no significant difference between two group in five frequency bands (all P-value > 0.01). The data were expressed as the means ± SEM.

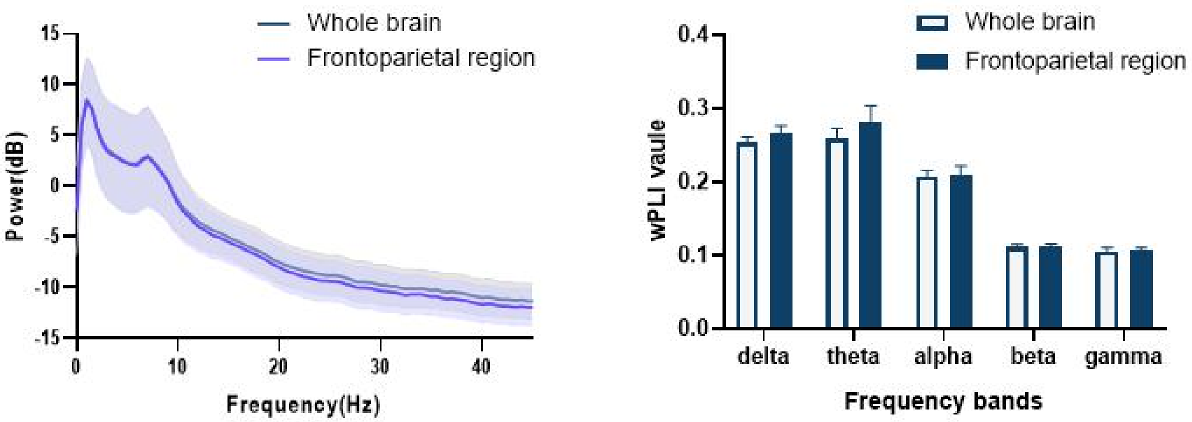

Supplementary Figure 3. Global and frontoparietal changes in the EEG power spectra and functional connectivity in EMCS group. (A) The average absolute EEG power in frontoparietal region (purple line) and whole brain (blue line). The Y-axis represented the log-transformed power. (B) Box plot of wPLI values between frontoparietal region and whole brain. There was no significant difference between two group in five frequency bands (all P-value > 0.01).
